# Recent developments in the Inorganic Crystal Structure Database: theoretical crystal structure data and related features

**DOI:** 10.1107/S160057671900997X

**Published:** 2019-09-23

**Authors:** D. Zagorac, H. Müller, S. Ruehl, J. Zagorac, S. Rehme

**Affiliations:** a Technicum Scientific Publishing, Stuttgart, Germany; b Institute of Nuclear Sciences Vinča, Materials Science Laboratory, Belgrade University, Belgrade, Serbia; c FIZ Karlsruhe – Leibniz Institute for Information Infrastructure, Karlsruhe, Germany

**Keywords:** Inorganic Crystal Structure Database, ICSD, theoretical structures, standardization, classification

## Abstract

The Inorganic Crystal Structure Database (ICSD) is the world’s largest database of fully evaluated and published crystal structure data, mostly obtained from experimental results. However, the purely experimental approach is no longer the only route to discover new compounds and structures. In the past few decades, numerous computational methods for simulating and predicting structures of inorganic solids have emerged, creating large numbers of theoretical crystal data. In order to take account of these new developments the scope of the ICSD was extended in 2017 to include theoretical structures which are published in peer-reviewed journals. Each theoretical structure has been carefully evaluated, and the resulting CIF has been extended and standardized. Furthermore, a first classification of theoretical data in the ICSD is presented, including additional categories used for comparison of experimental and theoretical information.

## Abstract 2

The article discusses how theoretical crystal data are supplementing experimental data for simulation and prediction of structures of inorganic solids in the Inorganic Crystal Structure Database.

## 1. Introduction   

The Inorganic Crystal Structure Database (ICSD) contains an almost exhaustive list of known inorganic crystal structures published since 1913 (Bergerhoff & Brown, 1987 ▸; Belsky *et al.*, 2002 ▸). In particular, the database provides information on structural data of pure elements, minerals, metals and intermetallic compounds. In order to be included in the database, a structure has to be fully characterized, the atomic coordinates determined and the composition fully specified. A typical entry includes, *inter alia*, the chemical name, formula, unit cell, space group, complete atomic parameters (including atomic displacement parameters), site occupation factors, title, authors and literature citation. In addition to the published data, many items are added through expert evaluation or are generated by computer programs, such as the Wyckoff sequence, molecular formula and weight, ANX^1^ formula, mineral group *etc*. (Buchsbaum *et al.*, 2010 ▸). Of course, full bibliographic information is also included; for newer entries often even the abstract is provided.

All crystal structures contained in the database have been carefully evaluated and checked for quality related to formal errors and scientific accuracy by our expert editorial team. We continuously extract and abstract the original data from over 80 leading scientific journals and an additional 1300 scientific journals. The ICSD is updated twice a year, each time adding approximately 4000 new records. As the size of the ICSD has grown over time, we have continuously enhanced the quality of our data. At present (2018.2 release), the ICSD contains more than 200 000 entries, including 2902 crystal structures of the elements, 38 506 records for binary compounds, 73 048 records for ternary compounds, and 73 688 records for quarternary and quintenary compounds. About 159 000 entries (80%) have been assigned to one of 9015 structure types (Allmann & Hinek, 2007 ▸). The remaining 20% of entries are not assigned to any existing structure type, as such compounds would be individual compounds with a new structure type of their own and, according to our definition, a structure type has to contain at least two compounds.

**Figure 1:**
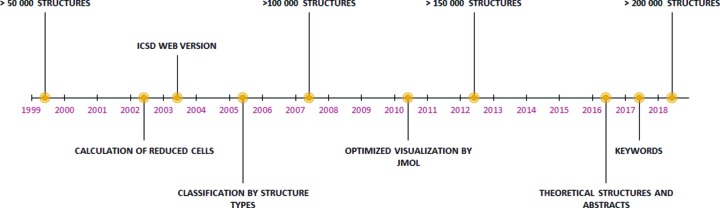
ICSD timeline.

In the beginnings of the ICSD, the focus was merely on collecting and editing data. The data available in the literature were identified and examined according to defined quality criteria. In the meantime, the ICSD has evolved from a mere collection of data into a versatile tool for research and materials science (Fig. 1 ▸). Pure structure information is combined with information on physical–chemical properties and measurement methods. This means that the data can be used more universally. Last but not least, as a result of discussions about data mining and the application of semantic tools, article- and structure-related keywords (not necessarily identical to the author keywords, which are often too general) and the abstracts contained in the articles have been included in the database in recent years. Starting with the publication year 2015, theoretical (calculated) structures have also been recorded in the ICSD.

A database needs to cover several essential aspects in order to be useful (Buchsbaum *et al.*, 2010 ▸). The first aspect is the comparability of data. For crystallographic data this is easy as the comparability is already based on the principles of crystallography itself and further enforced by standardizing all crystal structures for better comparison. A generally accepted format is even defined for the exchange of crystallographic information (crystallographic information file – CIF; Hall, 1991 ▸; Hall & Spadaccini, 1994 ▸). The second important aspect is the completeness of data. Statistical interpretations based only on a small subset will probably not produce results with a high level of significance. The last and most decisive factor is the quality of the data. Unreliable data can only lead to unreliable results. For the ICSD, in the case of distinctive features the author is contacted or a remark is set.

## 2. Comparison with other crystal-structure-based databases   

In addition to the ICSD, there are several other commercial and non-commercial structure-based databases. Among the commercial databases, the Cambridge Structural Database (CSD; Groom *et al.*, 2016 ▸) published by the Cambridge Crystallographic Data Centre (CCDC), the various powder diffraction file (PDF; ICDD, 2018 ▸) databases of the International Centre for Diffraction Data, Pearson’s Crystal Data (Villars & Cenzual, 2018 ▸), CrystMet (White *et al.*, 2002 ▸) and AtomWork-Adv (NIMS, 2018 ▸) are worth mentioning.

The bandwidth of non-commercial databases is very wide, ranging from the Protein Data Bank (Berman *et al.*, 2000 ▸), which specializes in proteins and nucleic acids, to the generally oriented Crystallography Open Database (Gražulis *et al.*, 2012 ▸) and the American Mineralogist Crystal Structure Database (Downs & Hall-Wallace, 2003 ▸), to a large number of databases for calculated structures (Curtarolo *et al.*, 2012 ▸; Jain *et al.*, 2013 ▸; Saal *et al.*, 2013 ▸; Draxl & Scheffler, 2018 ▸; http://openmaterialsdb.se/; Ortiz *et al.*, 2009 ▸).

Table 1 ▸ bundles the most important information of the different databases and allows quick comparison.

Apart from the differences in domain coverage and some special functionalities, the completeness and consistency of the experimental data offered is certainly the greatest in the commercial services. For the mentioned purposes of data mining these databases are most suitable.

FIZ Karlsruhe has been cooperating with the CCDC since 2017 and provides a joint crystal structure depository in which all crystal structures of the CSD, the ICSD and the previous separate crystal structure depositories are stored and freely accessible – however, the search options are very limited and the export of crystal structures is also limited. Further cooperations are planned and will bring the two databases even closer together.

## 3. Keywords in the ICSD   

Compounds with defined material properties can be searched in the ICSD owing to the introduction of keywords. Search results based on titles and abstracts are often limited, because they present the author’s priorities. Specific keywords are assigned according to the content of the article and are therefore more precise. In most cases an article already contains author keywords. These, however, are often too general (*e.g.* ‘crystal structure’) and not suited for searching for defined properties and methods. The keywords in the ICSD are assigned according to a defined thesaurus and thus standardized. Additional free-text entries can be made in exceptional cases.

At the beginning of the assignment of keywords, about 20 000 keywords, mainly from the fields of magnetism and spectroscopy, were assigned to about 6000 journal articles from the running production within the year 2018.

As regards the frequency distribution of the individual keywords, almost 280 keywords (as per January 2019) were considered relevant and made available to the users as a first *ad hoc* list: not least in order to receive feedback from the users as soon as possible.

The current ICSD thesaurus is not static but is continuously extended. Depending on the development of the discipline we will see where a deeper indexing will be required in the future. Among the next steps – besides the desired feedback from the community – could be a comparison with recognized thesauri and ontologies from science and technology in order to close gaps in the hierarchic structure of the ICSD. We also plan to employ data mining procedures in order to index ICSD structures retrospectively on the basis of titles and abstracts.

**Figure 2:**
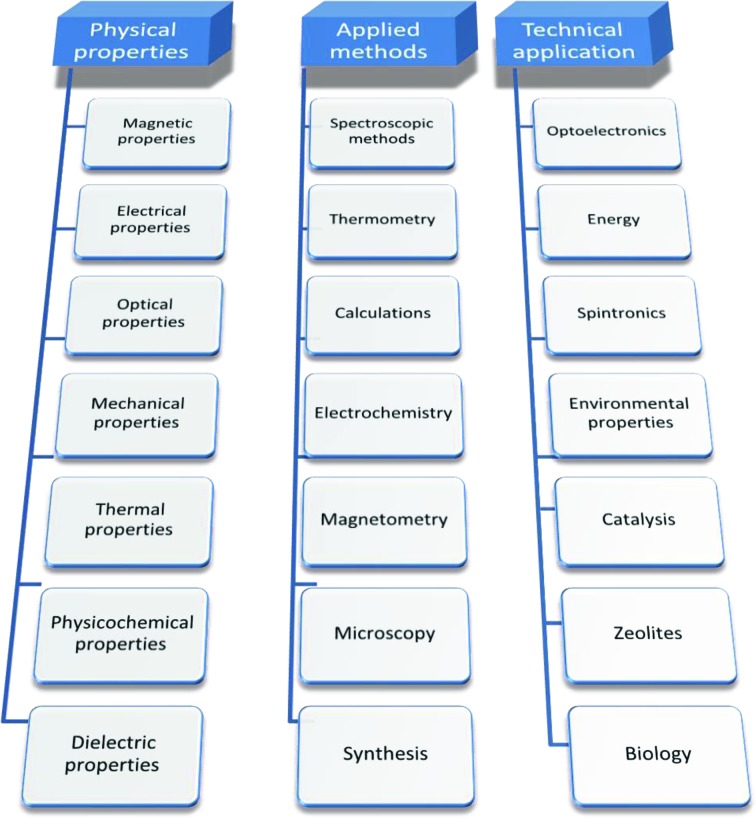
Set of predefined keywords standardized according to physical properties of materials, applied methods and technical application, fully searchable in the ICSD. For the full list of the standardized keywords see the supporting information.

ICSD keywords describe material properties, analysis methods used or technical fields of application (Fig. 2 ▸).

In particular, material properties are further classified as magnetic properties, electrical properties, optical properties, mechanical properties, thermal properties, physicochemical properties and dielectric properties (Fig. 2 ▸). Each of these rather broad descriptions is further split into more detailed keywords fully searchable in the ICSD, *e.g.* magnetic properties with magnetic susceptibility or ferromagnetism, electrical properties with superconductivity or piezoelectricity, and so on (for more details see the supporting information).

Similarly, applied methods are also classified into spectroscopic methods, thermometry, calculations, electrochemistry, magnetometry, microscopy, crystal structure, chemical composition^2^ and synthesis, and for each of them specific keywords are assigned which are frequently encountered in scientific and industrial work (for details *cf*. supporting information). Technical application is described by the keywords optoelectronics, energy, spintronics, environmental properties, catalysis, zeolites and biology. As in previous cases, a set of more detailed keywords has been assigned, fully searchable in the ICSD, *i.e.* for optics keywords like nonlinear optics (NLO) materials or light emitting diode (LED) technology, for energy keywords like solar cells or batteries, and so on (*cf*. supporting information). In addition to standardized keywords, a free-text keywords search is available in the ICSD, *e.g.* in order to search the ICSD for nanostructures, the user needs to type free-text nano (*e.g.* Káňa *et al.*, 2016 ▸; Miao *et al.*, 2016 ▸).

In summary, the use of keywords combined with, for example, chemical (elements) or structural (structure types) information easily enables searches for special materials like superconductors or piezoelectric materials or technical applications like solar cells or solid electrolytes.

## 4. Theoretical structures   

### 4.1. Standardization of theoretical crystal structures in the ICSD   

**Figure 3:**
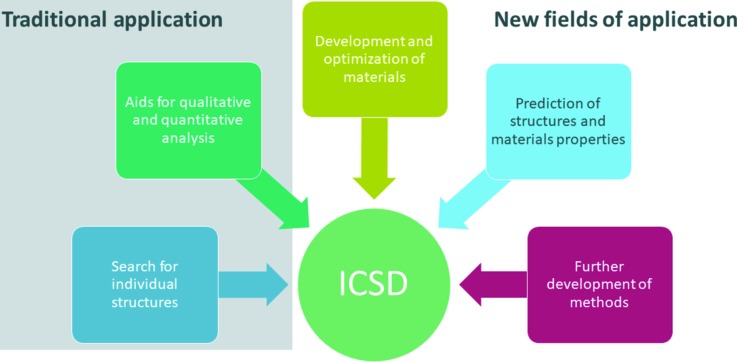
ICSD application graph, going from traditional applications such as searches for individual structures and using them in qualitative or quantitative analysis, to new fields of application, where the data are used to develop or optimize new materials following either the classical synthesis approach or the more modern *in silico* approach.

More and more tailor-made materials with predefined properties are being produced (Butler *et al.*, 2016 ▸). Predicting material properties or synthesizing special properties can save time-consuming and expensive work in the laboratory, and this is now possible because of the availability of high computing power and improved computer programs (Curtarolo *et al.*, 2013 ▸). To develop new materials, it is usually necessary to use structure information from existing and already measured compounds contained in suitable databases, *e.g.* the ICSD. Optimizing the available parameters or comparing measured and calculated results can then lead to new conclusions, as summarized in Fig. 3 ▸.

The ICSD is already extensively used in data mining and in computational chemistry. The traditional approach in materials research of first synthesizing new compounds and then checking their properties is rather time consuming and quite expensive. One can already observe a strong tendency to shift materials research from the traditional synthesis-oriented approach to a more theory-oriented approach. On the other hand there are numerous problems with available theoretical data (lack of file format standardization, the variety of methods/codes *etc*.), and perhaps the major problem is the huge quantity of calculated data with a broad variety of quality.

In order to tackle these problems, we have performed data standardization of the theoretical crystal structures. Data standardization is the critical process of bringing data into a common format, implementing and developing technical standards, and helping to maximize the quality of the data. In particular, a set of selection criteria has been developed in order to standardize theoretical crystal structure data. We have three major criteria for the selection of theoretical structures:

(*a*) publication criterion;

(*b*) total energy criterion;

(*c*) multiple methods criterion.

The first criterion for selection of theoretical structures is publication in a peer-reviewed journal. In this way, we are able to discard a large quantity of theoretical data which are unpublished and stored in various databases, with unknown origin or quality. However, this selection criterion involves careful evaluation and a great amount of manual work through inspection of individual research papers in order to ensure high quality of the extracted theoretical structures. The second criterion includes total energy ranking of the theoretical structures. In principle, theoretical structures which have low total energy are considered to be close to the equilibrium structure and suitable for storing in the ICSD. In addition, theoretical structures that are affected by external conditions (pressure, temperature, magnetic field *etc*.) are deposited, and this information is provided in the corresponding CIF. Similarly, theoretical structures with negative formation energies are considered suitable. In this way a large number of high-energy and extremely metastable theoretical structures, which are not likely to be synthesized, are excluded. The final criterion is applicable when multiple theoretical methods have been applied to calculate the same starting structure. In such cases, the theoretical method which delivers data closest to the corresponding experimental results is chosen for storing in the ICSD, while other methods applied are only noted as a comment.

Otherwise, theoretical data are completely coherent with experimental data in the CIF: for example, each record contains information on compounds which have no C—C and/or C–H bonds and which include structural data of pure elements, minerals, metals and intermetallic compounds; structural descriptors (Pearson symbol, ANX formula, Wyckoff sequences); and bibliographic data. In order to be included in the ICSD, a theoretical structure has to be fully characterized, the atomic coordinates determined and the composition fully specified, similarly to experimental structures. Each of the theoretical crystal structures contained in the database has been carefully evaluated and checked for quality by our expert editorial team.

### 4.2. Classification and categorization of theoretical data in the ICSD   

**Figure 4:**
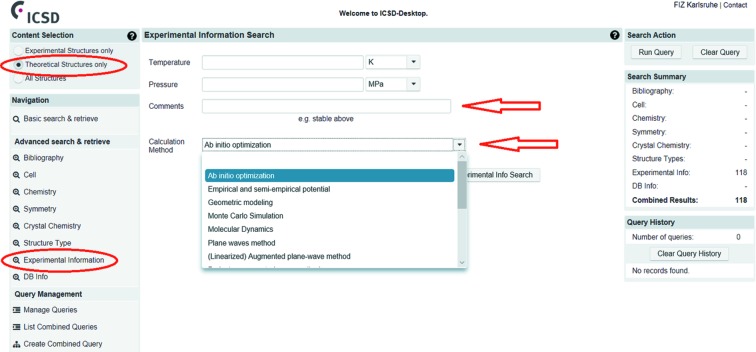
In the ‘Content Selection’ the user can choose ‘Theoretical Structures only’ (upper left corner) and afterwards ‘Experimental Information’ in the bottom left corner. The user is now directed to the ‘Experimental Information Search’ section in the middle, where user can choose one of the theoretical categories in the ‘Calculation Method’ field (bottom arrow). In the upper ‘Comments’ field, the user can search the ICSD for technical details of the calculations (upper arrow).

In this section, a novel classification and categorization of theoretical data in the ICSD will be presented. Theoretical crystal structures are labelled in the ICSD to allow an easy distinction between theoretical and experimental structures (Fig. 4 ▸). The user also has the option to include all structures in a search (for a detailed description see the supporting information). Furthermore, we have defined a set of keywords which are specific for theoretical structures and which describe, for example, the methods or the details of the calculation. This ensures that the user will be able to select and evaluate those structures in a very precise manner.

In total we have defined 13 categories which correspond to the theoretical methods used to calculate theoretical crystal structures (see Table 2 ▸). Although these categories are relevant mostly for the growing field of theoretical studies, the final benefit should be for all users of the ICSD. In that respect we have suggested several theoretical categories, which are found to be most popular in the papers published with theoretical structures in the ICSD, which include the choice of the energy (cost) function, mathematical modelling, quantum chemical methods and functionals. For example, *ab initio* optimization, or empirical and semi-empirical potential, stands for calculations performed using the respective potentials, while geometric modelling is used when theoretical structures are obtained using mathematical and/or crystallographic models. We note that the augmented plane-wave method includes the full-potential (linearized) augmented plane-wave [(L)APW] + local orbitals (lo) method, while the linear muffin-tin orbital (LMTO) method also includes the full-potential (FP)–LMTO–atomic spheres approximation (ASA).

These theoretical categories are very useful tools for all users of the ICSD, but above all theoreticians. Possible applications span from statistics in the specific theoretical category and potential use for future calculations, to data mining and method development. In addition to these 13 theoretical methods, we provide further classification and categorization based on information obtained from comparison of theoretical and experimental structures. The first such category is ‘predicted (non-existing) crystal structure’ (Table 2 ▸). As crystal structure predictions become more and more reliable, this category can be an excellent tool for synthesis planning.^3^ In particular, obtaining information on not-synthesized unknown compounds or/and not-synthesized modifications of known compounds could be an important advantage for ICSD users with numerous scientific, technological and industrial applications. The next category is ‘optimized (existing) crystal structure’, which compares the optimized theoretical structure with all existing experimental crystal structures in the ICSD until the year of publication. Optimized structures can also be an excellent tool for various applications: for instance, applications in computational materials science and related sciences, where optimized structures can be used to generate parameters for future calculations. In experimental materials science and related sciences, they can be used as an excellent tool for industrial and technological applications where it is very important to fine-tune materials, because slight deviations between the calculation and experiment can lead to different properties of the material. This can be even further examined by combining optimized structures with standardized keywords for physical properties. The final category is ‘combination of theoretical and experimental structure’. If such data exist in the manuscript they are highly valuable to all materials scientists with a great variety of possible applications, owing to the high precision of the published data.

These categories allow comparison of calculated structures either with each other or directly with experimental data, making the categorization a useful tool in both experimental and computational materials science. Together with the previous theoretical methods, this makes in total 16 theoretical categories in the ICSD, and a complete summary of these categories is shown in Table 2 ▸.

Finally, the ICSD provides additional computational information used in the calculation of the respective theoretical crystal structures. This computational information provides details about the code, search algorithm, method, basis set information and technical details of the calculation (*e.g.* cutoff energy, K-point mesh *etc.*), providing information on reproducibility and quality of computations. In addition we provide comments on the tolerances in energy, forces *etc*. used in calculations if present (which are similar to the experimental structure criteria *R* factors, FOMs *etc*.). If the theoretical structure is missing the total energy criterion, meaning that the manuscript does not provide total energies, formation energies *etc*., the comment ‘Etot ranking is missing in the paper’ is added (corresponding to the ‘*R* factors are missing in the manuscript’ comment). If there exist more structures in the manuscript but they are, say, energetically high and unstable, the comment ‘Additional structures are published in the manuscript’ is included. Furthermore, if the theoretical structure shows magnetic properties, we add comments about the magnetic state, inclusion of spin orbit interaction *etc*., which provide additional information on the quality of the calculation. Finally, we provide information about the code used to calculate the theoretical structure, and if additionally another code has been used, for example, for electronic property calculation or phonon calculations. Since this information is fully searchable in the ICSD, it can be a very useful tool for future theoretical studies.

## 5. Applications of the ICSD   

### 5.1. Discovery of new ionic conductors and solar cell absorber   

An example of using crystallographic data to predict material properties is the systematic identification of new possible Na-ion conductors in ternary Na oxides for replacing Li in batteries. For this, Meutzner *et al.* (2015 ▸, 2017 ▸) applied the Voronoi–Dirichlet approach and were able to identify around 50 high-potential candidates for solid ionic conductors from several thousand possible structures.

The mutual influence of theoretical and experimental data in the ICSD can be illustrated by another example, where the potentially stable structure and properties of wurtzite, CuGaO_2_, were calculated via density functional theory (DFT) (Omata *et al.*, 2014 ▸). The subsequent synthesis and analysis of the compound confirmed the expected semiconductive properties (Nagatani *et al.*, 2015 ▸).

### 5.2. Prediction of novel advanced ceramic materials   

Aluminium nitride is an interesting semiconductor ceramic material with various technological and industrial applications. In this example study, data mining of over 140 000 structures in the ICSD has been performed, followed by *ab initio* optimizations (Zagorac *et al.*, 2017*b* ▸). Finally, 12 new structure candidates were proven to be the most promising ones, which later showed diverse electronic, elastic and mechanical properties (Zagorac *et al.*, 2018 ▸).

Similarly, transition metal silicides have attracted great attention owing to their potential applications in microelectronics, ceramics and the aerospace industry. In another example, experimental and theoretical investigations of tungsten-based silicides were performed, and new modifications were obtained using entries from the ICSD as starting points in the first principles calculations (Luković *et al.*, 2017 ▸).

### 5.3. Finding nature’s missing binary and ternary oxide compounds   

Finding new compounds and their crystal structures is an essential step in discovering new materials. In this example, low-enthalpy phases of TiO_2_ and SiO_2_ at extreme pressure conditions were calculated using DFT. It has been found that the most stable form of TiO_2_ at pressures above 650 GPa is a ten-coordinated structure with space group *I*4/*mmm*. TiO_2_ is the well established high-pressure model for many *AX*_2_ compounds, and this study showed that SiO_2_ should also form in the *I*4/*mmm* structure above 10 TPa (Lyle *et al.*, 2015 ▸).

In the next example a probabilistic model built on experimental data from the ICSD, novel compositions that are most likely to form a compound and their most probable crystal structures were identified and tested for stability by *ab initio* computations. A large-scale search for new ternary oxides has been performed, which resulted in the discovery of 209 new compounds (Hautier *et al.*, 2010 ▸).

### 5.4. Structural relations studies within the ICSD   

The ICSD is a very useful tool for investigating the structural relations between various inorganic crystalline compounds. Such a study has been performed for chemical systems listed in the ICSD using a geometry-based similarity criterion. By applying all entries in the ICSD to the structure comparison algorithm CMPZ, ordered crystalline structures contained in the ICSD were classified into structure families and their relations investigated (Sultania *et al.*, 2012 ▸). In the latter work, a hierarchical set of criteria for the separation of isopointal structures into isoconfigurational structure types has been used. It has been shown how these criteria, which include the space group, Wyckoff sequence and Pearson symbol, *c*/*a* ratio, β ranges, ANX formulae, and, in certain cases, the necessary elements and forbidden elements, may be used to uniquely identify the representative structure types of the compounds contained in the ICSD (Schön, 2014 ▸).

## 6. Conclusion   

The ICSD is already extensively used in computational and experimental materials science and related natural sciences. In particular, crystal structure predictions have become more and more reliable. This allows comparison of calculated structures either with each other or directly with experimental data. Here, we explain the introduction of theoretical CIFs into the ICSD. Each theoretical structure is extended and standardized and completely coherent with the structural standards used for experimental entries. We introduce the categorization of theoretical data in the ICSD. Finally, we present the connection of theoretical structures with material properties, applied methods and/or applications using the keyword option. This combination is an excellent tool for data mining. Therefore, the inclusion of theoretical data not only extends the scope of the ICSD significantly, it also allows data mining applications that were not possible previously while also increasing the range of data for more classical applications.

## Supplementary Material

Screenshot and keyword list. DOI: 10.1107/S160057671900997X/in5024sup1.pdf

## Figures and Tables

**Table 1:** Comparison of databases containing experimental and/or theoretical crystal structures

	No. of entries	Content	Remarks
ICSD	∼210 000	Inorganic and metal–organic compounds	Commercial, experimental and calculated structures, material properties
CSD	∼1 000 000	Organic and metal–organic compounds	Commercial, experimental structures
PDF	∼410 000 (PDF-4+)	Inorganic and organic compounds	Commercial, powder data, not all entries include atomic coordinates
Pearson’s Crystal Data	∼319 000	Inorganic compounds	Commercial, experimental structures, not all entries include atomic coordinates
CrystMet	∼180 000	Inorganic compounds	Commercial, experimental structures
AtomWorks-Adv	∼300 000	Inorganic compounds	Commercial, experimental structures and material properties, not all entries include atomic coordinates
Protein Data Bank	∼150 000	Proteins, nucleic acids	Open access, experimental structures
Crystallography Open Database	∼400 000	Inorganic and organic compounds	Open access, experimental structures
American Mineralogist Crystal Structure Database	∼20 000	Only minerals	Open access, experimental structures
Aflowlib	∼2 800 000	∼350 000 binaries, ∼1 900 000 ternaries and ∼450 000 quaternaries	Open access, calculated structures and material properties, all calculated using Aflow
Materials Project	Unknown	∼530 000 nano-porous compounds, ∼130 000 inorganic compounds	Open access, calculated structures and material properties
Open Quantum Materials Database	∼560 000	Inorganic compounds	Open access, calculated structures and material properties
Nomad	∼50 000 000	Inorganic and organic compounds	Open access, calculated structures and material properties
Open Materials Database	∼200 000	Inorganic and organic compounds	Open access, calculated structures and material properties
Electronic Structure Project	∼60 000	Inorganic and organic compounds	Open access, calculated structures
			

**Table 2:** Summary of theoretical categories in the ICSD

Theoretical category in the ICSD	References†
*Ab initio* optimization	Zagorac *et al.* (2014*a* ▸); Mayo *et al.* (2016 ▸)
Empirical and semi-empirical potential	Fan *et al.* (2015 ▸); Yoo *et al.* (2016 ▸)
Geometric modelling	Zagorac *et al.* (2014*b* ▸); George *et al.* (2015 ▸)
Monte Carlo simulation	Hao *et al.* (2014 ▸); Mena *et al.* (2016 ▸)
Molecular dynamics	Schmidt *et al.* (2015 ▸); Paściak *et al.* (2015 ▸)
Plane waves method	Weerasinghe *et al.* (2015 ▸); Goncharov *et al.* (2016 ▸)
FP(L) augmented plane-wave method (+lo)	Mukadam *et al.* (2016 ▸); Čebela *et al.* (2017 ▸)
Projector augmented wave method	Zurek & Yao (2015 ▸); Buckeridge *et al.* (2016 ▸)
Linear combination of atomic orbitals method	Zagorac *et al.* (2011 ▸); Larbi *et al.* (2016 ▸)
(FP) linear muffin-tin orbital (ASA)	Uba *et al.* (2016 ▸); Mishra & Ganguli (2016 ▸)
Hartree–Fock method	Shimazaki & Nakajima (2015 ▸); Zagorac *et al.* (2017*a* ▸)
Density functional theory	Civalleri *et al.* (2007 ▸); Schönecker *et al.* (2015 ▸)
Hybrid functionals	Lee *et al.* (2015 ▸); Sluydts *et al.* (2017 ▸)
Predicted (non-existing) crystal structure	Doll *et al.* (2008 ▸); Luković *et al.* (2017 ▸)
Optimized (existing) crystal structure	Olsson *et al.* (2015 ▸); Erba *et al.* (2015 ▸)
Combination of theoretical and experimental structure	Retuerto *et al.* (2016 ▸); Cvijović-Alagić *et al.* (2019 ▸)

† References to example theoretical structures found using that theoretical method and already searchable in the ICSD.

## Footnotes

1 The ANX formula is a simple classification for structures based on the oxidation states of the elements involved. Elements with a positive oxidation state are identified by the first letters of the alphabet A–M, elements with a negative oxidation state by the last letters of the alphabet S–Z and elements with an oxidation state of 0 by the letters N–R. The letters are sorted by increasing index (AB2X4, not A2BX4). Structures containing more than four positive, three negative or three neutral atomic types are not considered. The ANX formula is only calculated for completely determined structures and hydrogen atoms are not considered.

2 Not included in Fig. 2; for a current list of keywords see https://icsd.products.fiz-karlsruhe.de/en/howuse/how-use.

3 Of course, we note the time restriction of the predicted structures, due to the publication year. On the other hand, many theoretical structures remain non-synthesized many years after publication.
