# Supplementary material for: Recent developments in the Inorganic Crystal Structure Database: theoretical crystal structure data and related features
Source: J Appl Crystallogr. 2019 Sep 23;52(Pt 5):918–25. doi: 10.1107/S160057671900997X (PMC6782081; doi:10.1107/S160057671900997X)
Supplement: Supplementary file 1 [file j-52-00918-sup1.pdf]

Recent developments in the ICSD database: theoretical crystal structure data and related features

D. Zagorac[1,2], H. Müller[3], S. Rühl[3], J. Zagorac[1,2], S. Rehme[3]

[1]Technicum Scientific Publishing, Stuttgart, Germany
[2]Institute of Nuclear Sciences Vinča, Materials Science Laboratory, Belgrade University, Belgrade, Serbia
[3]FIZ Karlsruhe – Leibniz Institute for Information Infrastructure, Karlsruhe, Germany

Supporting Information

1. Example of the ICSD desktop

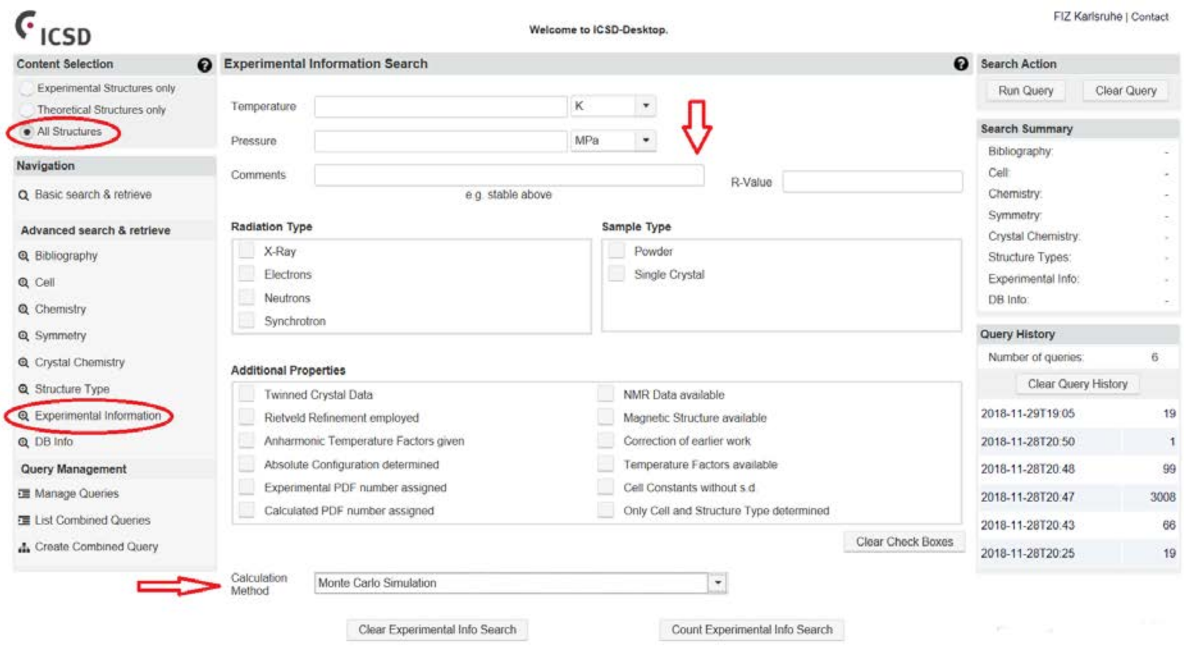

Fig. S1. If one chooses *All Structures* in the Content Selection (upper left corner), the interface shows more information in the *Experimental Information Search* mask. In addition to the information for the theoretical structures some more information about experimental structures is now included.

2. The complete list of standardized keywords in the ICSD database (November 2018)

- Physical properties
  - Magnetic properties
    - Antiferromagnetism
    - Diamagnetism
    - Ferrimagnetism
    - Ferromagnetism
    - Hysteresis
    - Magnetic dynamics
    - Magnetic exchange interactions
    - Magnetic frustration
    - Magnetic moment
    - Magnetic ordering
    - Magnetic refrigeration
    - Magnetic structure
    - Magnetic susceptibility
    - Magnetization
    - Magnetization dynamics
    - Magnetization relaxation
    - Magnetostriction
    - Magnetocaloric effect
    - Magnetoelectric properties
    - Multiferroic
    - Paramagnetism, Superparamagnetism
    - Spin-chain systems
    - Spintronic material
  - Electrical properties
    - Dielectric permittivity
    - Dielectric constant
    - Electrical conductivity
    - Electrical resistivity
    - Electrochemical properties
    - Ferroelectricity
    - Hall effect
    - Ionic conductivity
    - Magnetoelectric properties
    - Piezoelectric constant
    - Piezoelectricity
    - Proton conduction
    - Semiconductor
    - Superconductivity
    - Superionic conduction
    - Thermoelectric properties
  - Optical properties
    - Birefringence

- CIE chromaticity coordinates
- Fluorescence
- Luminescence
- Mechanochromic behavior
- Non-linear optical response
- Photoactivation
- Photoluminescence (PL)
- Photonic material
- Photorefractivity
- Photosensitivity
- Reflectivity
- Refractive index
- Second harmonic generation (SHG)
- Transparency

Mechanical properties

- Bimetallic
- Bulk modulus
- Compressibility
- Elastic constants
- Memory-shape crystal
- Piezoelectricity
- Shear modulus
- Ultra-sonic
- Vickers hardness

Thermal properties

- Anisotropic thermal expansion
- Heat capacity
- Negative thermal expansion
- Seebeck effect
- Specific heat
- Thermal conductivity
- Thermal expansion
- Thermochromism
- Thermoelectric properties

Physicochemical properties

- Bivalence
- Catalytic activity
- Electrochemical properties
- Kinetic parameters
- Mixed valence
- Sensitivity toward impact and friction
- Stability constants
- Thermodynamic properties

Dielectric properties

- AC electrical conductivity
- DC electrical conductivity

- Dielectric constant
- Nyquist plots

Applied methods

- Spectroscopic methods
  - Dielectric spectroscopy
  - ESR
  - EXAFS
  - FTIR
  - Impedance spectroscopy
  - IR
  - Moessbauer
  - NMR
  - NQR
  - Phonon-modes analysis
  - Raman
  - UV/vis
  - UV/vis optical absorbance spectra
  - UV/vis-NIR emission spectroscopy
  - UV/vis-NIR reflectance spectroscopy
  - UV/vis-NIR spectroscopy
  - Vibration-modes analysis
  - X-ray absorption spectroscopy (XAS)
  - X-ray photoelectron spectroscopy (XPS)
  - XANES
- Calculation methods
  - ab initio calculations
  - Band structure
  - BVS
  - Computational studies
  - DFT
  - DOD, COHP
  - Electronic structure calculations (e.g. DOS)
  - Geometry optimization
  - MAPLE calculations
  - Molecular orbital calculations (MO)
  - Monte Carlo calculations
  - Natural bond orbital analysis (NBO)
  - QTAIM calculations
  - Quantum-chemical calculations
  - TD-DFT
  - Thermodynamic calculations
  - Valence bond analysis
- Thermometry
  - Differential scanning calorimetry (DSC)
  - Differential thermal analysis (DTA)
  - Thermal analysis

Thermal stability
Thermogravimetry (TGA,TG)

Electrochemistry
- Charge/Discharge curves
- Cyclovoltammetry (CV)
- Potentiometry
- Redox properties
- Solid electrolytes

Magnetometry
- SQUID magnetometry

Microscopy
- Atomic force microscopy (AFM)
- Contact Kelvin Probe Force Microscopy (cKPFM)
- Electron backscatter diffraction (EBSD)
- HAADF-STEM
- High resolution electron microscopy (HREM)
- Piezoresponse force microscopy (PFM)
- Scanning electron microscopy (SEM)
- Selected area electron diffraction (SAED)
- Transmission electron microscopy (TEM)

Crystal structure
- (3+1)D super space group
- Coordination environment
- Crystallography instrumentation
- Doping
- Electron backscatter diffraction (EBSD)
- Enantiomorphism, chiral structures
- Fourier maps
- Framework structure
- Group-subgroup relationship
- Hydrogen bond interactions
- Interstitial site
- Layered structures
- Li+ intercalation
- Lithiation
- Modulated structure
- Nanoparticles (NP)
- New mineral
- New structure type
- Phase diagram
- Phase transition
- Polymer structure
- Polymorphism
- Polytypism
- Quasicrystals
- Rotation electron diffraction (RED)

- Satellite peaks
- Selected area electron diffraction (SAED)
- Single-crystal growth
- Single-crystal to single-crystal transformation (SCSC)
- Stacking variants
- Superflip refinement
- Topology
- Twinning
- Vacancies
- Van-der-Waals interactions

Chemical composition
- Doping
- Electron micro probe analysis (EMPA)
- Energy-dispersive X-ray spectroscopy (EDX)
- Intermetallics
- Phase diagram
- Polyoxometalates (POM)

Synthesis
- Arc-melting
- Asymmetric synthesis
- Chemical vapor transport (CVT)
- Czochralski method
- Floating zone method
- Flux-growth
- HT-HP synthesis
- Hydrothermal synthesis
- Ionothermal synthesis
- Reaction coordinates
- Reaction mechanism
- Self-assembly
- Solid-state synthesis
- Solvothermal synthesis
- Synthesis, Molecular Structure, Reaction mechanisms

Technical Application

Optoelectronics
- Deep-ultraviolet transparent materials (DUV)
- Laser materials
- LED technology
- NLO materials
- OLED technology
- Scintillator(radiation detection)
- White-light emission (WLE)

Energy
- Batteries
- Fuel cells
- High-energy-density materials

- Hydrogen evolution
- Hydrogen storage
- Ionic conductor
- Optical band gap
- Photovoltaic
- Scintillators
- Semiconductor
- Solar cells
- Solid electrolytes
- Solid oxide fuel cells (SOFC)
- Superconductors
- Thermoelectrics

Environmental properties
- Actinide extraction
- Cadmium immobilization
- CO2 emission
- CO2 fixation/reduction
- Corrosion inhibition
- Environmental pollutants detection
- Explosives
- Ion exchange
- Nuclear fuel cycle
- Organic dyes adsorption/decomposition
- Radionuclide waste disposal

Catalysis
- Alcohol oxidation
- Alkylation catalysts
- Asymmetric catalysis
- Bond activation
- Catalyst precursors
- Deoxygenation
- Electrocatalytic activity
- Enantioselective catalysts
- Gas absorption
- Hydrogenation catalysts
- N2 fixation, N2 activation
- Oxygen reduction reaction (ORR)
- Photocatalysis
- Photoelectrochemical water-splitting
- Polymerization
- Redox properties
- Water oxidation
- Water-splitting

Spintronics
- Data storage
- Phase-change materials

Zeolites
- Ion exchange
- Molecular sieve
- Gas sorption

Biology
- Anti-cancer drugs
- Bio-catalysis
- Fungicides/Pesticides
- Pharmaceutics
- Photosynthesis
